# Relationship Between Diet Quality, Intestinal Permeability, and Gut Microbiota Features in Individuals with Obesity

**DOI:** 10.3390/nu18050775

**Published:** 2026-02-27

**Authors:** Sarah M. Eaton, Weiwen Chai, Olivia Moss, Edward C. Deehan, Victoria Texieira Reis, Ali Keshavarzian, Heather E. Rasmussen

**Affiliations:** 1 Department of Nutrition and Health Sciences, University of Nebraska-Lincoln, Lincoln, NE 68583, USA; seaton5@unl.edu (S.M.E.); victoriaestr@gmail.com (V.T.R.); 2 Clinical Nutrition, Rush University Medical Center, Chicago, IL 60612, USA; oamoss@ucdavis.edu; 3 Department of Food Science and Technology, University of Nebraska-Lincoln, Lincoln, NE 68588, USA; edeehan2@unl.edu; 4 Nebraska Food for Health Center, University of Nebraska-Lincoln, Lincoln, NE 68588, USA; 5 Division of Digestive Diseases and Nutrition, Department of Internal Medicine, Rush University Medical Center, Chicago, IL 60304, USA; ali_keshavarzian@rush.edu

**Keywords:** Healthy Eating Index of 2010 dietary guidelines (HEI-2010), Mediterranean Dietary Pattern (MDP), Dietary Approaches to Stop Hypertension (DASH), intestinal permeability, gut microbiota

## Abstract

**Background/Objectives:** This study examined relationships between diet quality, as determined using three a priori-defined dietary patterns (Healthy Eating Index of 2010 dietary guidelines [HEI-2010], Mediterranean Dietary Pattern [MDP], and Dietary Approaches to Stop Hypertension [DASH]), intestinal permeability, and features of the gut microbiota in a diverse, obese sample. **Methods:** This was a post hoc, cross-sectional study including 103 healthy, obese individuals (43.8 ± 11.3 years, BMI: 37.5 ± 6.1 kg/m^2^, 64.1% African American). Dietary intake was assessed using the Vioscreen food frequency questionnaire. Intestinal permeability was assessed via urinary sugar excretion and microbiota features were characterized using 16S rRNA gene amplicon sequencing. Relationships between dietary pattern adherence, intestinal permeability, and gut microbiota were assessed using correlation coefficients and a general linear model. **Results:** Higher dietary pattern scores correlated with lower levels of intestinal permeability measures such as 24 h urinary sucralose (HEI-2010: r = −0.33, *p* = 0.002; MDP: r = −0.31, *p* = 0.004; DASH: r = −0.38, *p* < 0.0001) and 24 h sucralose-to-lactulose ratio (HEI-2010: r = −0.23, *p* = 0.03; MDP: r = −0.32, *p* = 0.003; DASH: r = −0.24, *p* = 0.03). Fruit intake consistently correlated with lower intestinal permeability measures (*p* < 0.05) across all three dietary patterns. Higher DASH scores correlated with lower Proteobacteria (r = −0.28, *p* = 0.004) and higher Verrucomicrobia (r = 0.30, *p* = 0.002) phylum abundance. **Conclusions:** The current results suggest a potential role for diet quality in promoting intestinal health.

## 1. Introduction

The importance of following healthful dietary patterns for promoting health and longevity is well documented [1]. Because humans typically do not consume foods in isolation, there is a need to observe the effect of eating patterns (a combination of food components that reflect the overall quality of an individual’s diet) as a whole rather than single foods or food groups; this is recognized by the emphasis on dietary patterns in recent nutrition-based recommendations [2]. While many healthful dietary patterns have been identified, no single dietary pattern is considered superior based on health benefits. Established diet quality indices include the Healthy Eating Index (HEI), Mediterranean Dietary Pattern (MDP), and Dietary Approaches to Stop Hypertension (DASH). Adherence to these indices is linked to a decreased risk for cardiovascular disease (CVD), Diabetes, and Alzheimer’s disease [3,4,5].

The gut microbiota is a complex ecological and functional community that influences human host physiology and disease susceptibility [6]. The intestinal barrier, consisting of mucus, an epithelial barrier, lymphocytes, innate immune cells, and humoral elements, interacts with the gut microbiota and cells of the immune system to shape host health [7]. Disturbed intestinal permeability may result in a loss of intestinal homeostasis, functional impairments, systemic inflammation and potential development of disease [7]. Research from animal and in vitro studies suggests that both specific bacterial taxa and their metabolic byproducts play a role in regulating intestinal barrier function. For instance, short-chain fatty acids, produced through the fermentation of non-digestible carbohydrates, have been shown to enhance the expression of tight junction proteins [8,9]. Probiotic supplementation has been associated with improved barrier integrity [10,11,12] and function [13]. More broadly, adherence to standardized dietary patterns such as the HEI, MDP and DASH was associated with a healthier and more favorable gut microbiota composition [14,15]. However, evidence directly connecting dietary patterns to intestinal permeability remains limited.

Furthermore, previous studies suggest that gut microbial composition differs between obese and non-obese individuals [16], and that obesity is often associated with increased intestinal permeability [13]. However, the impact of adherence to healthful dietary patterns on gut health, particularly intestinal permeability in individuals with obesity, remains poorly studied. A clearer understanding of how diet and diet quality influence gut microbiota features and intestinal permeability in this population is needed, as such insights could inform the development of targeted dietary strategies. Leveraging baseline data from a randomized, controlled trial that assessed the efficacy of a synergistic symbiotic supplement [13], the current study examined relationships between diet quality, as determined using a priori-defined dietary patterns (Healthy Eating Index of 2010 dietary guidelines [HEI-2010], MDP through the Mediterranean Eating Pattern for Americans, and DASH), and features of the gut microbiota and intestinal permeability across individuals with obesity. We hypothesize that greater adherence to these healthful dietary patterns is associated with reduced intestinal permeability and a higher abundance of beneficial gut microbiota characteristics.

## 2. Materials and Methods

### 2.1. Participants

This study utilized baseline data from a randomized, controlled trial that systematically assessed the ecological and functional impacts of a synergistic symbiotic treatment with participants aged 18 to 65 years with a body mass index (BMI) of 30.0–40.0 kg/m^2^ [13]. Individuals were excluded from the study for the following reasons: (1) prior intestinal resection; (2) history of GI diseases (except for hiatal hernia, gastroesophageal reflux disease, and hemorrhoids); (3) severe renal disease or markedly abnormal liver function; (4) antibiotic use within the last 12 weeks prior to enrollment; (5) intolerance of aspirin; (6) regular use of aspirin; (7) excessive alcohol intake; (8) presence of chronic metabolic disease (symptomatic cardiovascular disease, insulin-requiring or uncontrolled diabetes, current active treatment of cancer); (9) consumption of probiotics, prebiotics, or symbiotics within the last 2 weeks prior to enrollment; and (10) lactose intolerance.

### 2.2. Study Design and Protocol

The study protocol of the randomized, controlled trial has been previously described (the trial was registered at clinicaltrials.gov, identifier NCT02355210, date: 4 February 2015) [13]. The following are the data collection procedures that are relevant to the current study. In brief, study data were collected over three study visits for each participant. At Visit 1, potential participants were screened for eligibility and provided written informed consent. Vitals and anthropometrics were completed, and blood was obtained for endotoxin and metabolic markers. Participants were instructed to collect stool samples for microbiome analysis in anaerobic bags before Visit 2 and deliver samples within 24 h if stored at −20 °C or within five hours if stored at room temperature. At Visit 2, study participants completed a food frequency questionnaire if not completed before the visit. Within one week, participants returned for Visit 3 to provide urine samples for baseline intestinal permeability measurement. The study protocol was approved by the Institutional Review Board at Rush University Medical Center and informed consent was obtained from all the participants. All procedures were conducted according to the principles expressed in the Declaration of Helsinki.

### 2.3. Derivation of Dietary Patterns

Participants’ dietary intake was assessed by the Vioscreen food frequency questionnaire (FFQ), which is an online, validated questionnaire originally designed to assess long-term dietary intake anywhere from 30 days to one year [17]. From a total of 20 food categories, participants selected specific foods eaten, how often these foods were consumed, and the serving size for each food item using branching logic to determine the questions presented.

The components of HEI-2010 and relevant subcomponents were included in the Vioscreen report. HEI-2010 total and component scores were calculated using the simple HEI scoring algorithm method [18]. For each HEI-2010 component, relevant dietary constituents were aggregated and standardized per 1000 kcal of energy intake, except for the fatty acid component, which was calculated as the ratio of the sum of monounsaturated and polyunsaturated fatty acids to saturated fatty acids. Each component value was then scored based on the HEI-2010 scoring standards [19]. The individual component scores were summed to derive the total HEI-2010 score. The HEI-2010 includes 12 components, nine adequacy components (fruit, whole fruit, vegetables, greens and beans, whole grain, dairy, protein food, seafoods and plant proteins, fatty acids) and three moderation components (refined grains, sodium and empty calories). HEI-2010 was used as data was collected before the HEI-2015 was integrated into the Vioscreen FFQ.

DASH (Dietary Approaches to Stop Hypertension) and MDP (Mediterranean Dietary Pattern) were derived from the food list data by compiling and quantifying Vioscreen-provided food items representative of each DASH and MDP component. DASH scores were determined using the validated Folsom method composed of 11 categories and a total maximum score of 11 [20]. The 11 components included (1) total grain intake, (2) whole grain intake, (3) vegetables, (4) fruits, (5) dairy foods, (6) meats, poultry, and fish, (7) nuts, seeds, and dry beans, (8) percent kcal from fat, (9) percent kcal from saturated fat, (10) sweets, and (11) sodium [20].

Accordance to the MDP was determined using the Mediterranean Eating Pattern for Americans (MEPA), an MDP screener validated in samples of individuals residing in the United States [21]. The MDP scoring was completed based on pre-determined cutoffs for accordance for each of the 21 items, with a score of 0 or 1 for not accordant and accordant, respectively. The 21 items included the following: (1) dark leafy green vegetables, (2) other vegetables, (3) peanuts and tree nuts, (4) avocado, (5) berries, (6) fruit other than berries, (7) olive oil, (8) butter and cream, (9) red and processed meat, (10) poultry, (11) fish, (12) milk and yogurt, (13) full-fat cheese and cream, (14) beans and legumes, (15) whole grain bread, pasta, and cereal, (16) pastries, cookies, cakes, candy bars, and frozen desserts, (17) fast food, (18) pre-prepared and prepackaged boxed, canned, or frozen meals or snacks, (19) sugar sweetened beverages, (20) unsweetened beverages, and (21) alcohol.

### 2.4. Quantification of Intestinal Permeability

Participants were asked to empty their bladders and ingest a sugar mixture containing 2 g mannitol, 7.5 g lactulose, 40 mg sucrose, and 2 g sucralose after a 12 h overnight fast. Participants collected urine into three separate containers for 5 h, 7 h (after the 5 h collection), and 12 h (after the 7 h collection), for a total collection time of 24 h as described previously [22]. Urine volumes were recorded, and urine samples were analyzed for concentrations of mannitol, lactulose, sucralose, and sucrose using gas chromatography according to the protocol detailed elsewhere [22]. Intestinal permeability was expressed as a percentage of the oral dose excreted in the urine. Two urine collections were recorded: 0–5 h and 0–24 h. Five-hour (5 h) urinary lactulose, mannitol, sucrose, sucralose, and sucralose-to-lactulose ratio are primarily markers of small bowel permeability; and 24 h (24 h) urinary sucralose, lactulose, and sucralose-to-lactulose ratio excretion are markers of total gut permeability [23].

### 2.5. Analysis of Fecal Microbiota

Fecal samples were stored in aliquots at −80 °C until analysis. To characterize the gut microbiota, 16S rRNA gene amplicon sequencing (Illumina MiSeq platform v3 kit producing 300-bp paired-end sequences) was performed at the University of Minnesota Genomics Center, with all samples being included in a single run. As previously described [24], the V5-V6 region of the 16S rRNA gene was amplified using primer pair 784F (5′-RGGATTAGATACCC-3′) and 1064R (5′-CGACRRCCATGCANCACCT-3′). The generated sequences were quality-filtered using Illumina software, resulting in more than 96% of the samples meeting all quality control criteria. Samples that did not meet quality filtering criteria were removed from the analysis. All reads were trimmed to 240 base pairs using the FASTX-Toolkit. The reads were then merged and analyzed for their sequencing depth. Samples exceeding 37,000 reads were subsampled using Mothur v.1.31.162, while samples less than 37,000 sequences were not subsampled. This normalization approach was suggested by Weiss et al. to account for differences in the library size and minimize potential biases due to sequencing depth across samples [25]. Subsequently, reads were filtered by length with a minimum of 240 base pairs and a maximum of 260 base pairs, dereplicated, and clustered by operational taxonomic unit (OTU), and chimeras were removed and taxonomically assigned as previously described [24].

### 2.6. Analysis of Fecal Short-Chain Fatty Acids (SCFAs)

Fecal samples were analyzed for SCFAs as previously described [13]. In brief, the stool was centrifuged (12,000 rpm × 5 min), and a mixture of formic acid (20%), methanol, and 2-ethyl butyric acid (internal standard, 2 mg/mL in methanol) was added to the supernatant. A 0.5 mL sample was injected into a gas chromatography column (Stabilwax-DA, length 15 m, inner diameter 0.53 mm, film thickness 0.1 mm; Varian Chrompack, Bergen op Zoom, The Netherlands) in a Chrompack CP9001 gas chromatograph using an automatic sampler (Chrompack liquid sampler CP9050; Varian Chrompack). Peak areas for acetate, propionate, butyrate, and the branched-chain fatty acids isobutyrate and isovalerate relative to 2-ethyl butyric acid were quantified.

### 2.7. Statistical Analysis

Participants were categorized as high-dietary-adherence (HA, ≥median) and low-dietary-adherence (LA, <median) based on the median score for each dietary pattern. Baseline characteristics for participants categorized as HA were compared to those characterized as LA for each dietary pattern using the *t*-test for continuous variables and chi-square test for categorical variables. The scores derived from individual food component intake in each dietary pattern were compared between LA and HA using a *t*-test. Due to the relatively small sample size, we dichotomized the adherence scores for each dietary pattern.

The associations between dietary pattern scores and intestinal permeability markers were tested using partial correlation coefficients. The covariates included in the model were participant age (continuous), sex, race/ethnicity (African American, white, or other), education (less than high school, high school/some college, or college graduate), BMI (continuous), smoking status (current smoker or not), frequency of alcohol consumption (non-user, rare user, occasional user, or ≥1 drink/week), total daily energy intake (continuous), and metabolic syndrome score (score of 0 to 5 with a score of 1 for each of the five metabolic syndrome components based on pre-defined criteria [13,26]). To confirm the results from partial correlation coefficient analysis, we also compared permeability measures between HA and LA using a general linear model (GLM) adjusting for covariates mentioned above. The associations between key dietary components and key permeability measures (24 h urinary sucralose and the 24 h sucralose-to-lactulose ratio) were also assessed using partial correlation coefficients and GLM adjusting for covariates.

Spearman’s rank correlation coefficient was used to explore the relationships between dietary pattern scores and microbiota features and relationships between intestinal permeability measures and microbiota features. We aimed to determine whether any observed associations between dietary pattern scores and intestinal permeability could be partially explained by gut microbiota. All statistical modeling was conducted using SPSS (version 28, IBM Corp, Armonk, NY, USA). A *p* value <0.05 was considered statistically significant. For associations with microbiota features, *p* values were adjusted with the false discovery rate (FDR). The FDR was applied exclusively to microbiota variables due to the unique characteristics of microbiota data, such as compositionality and high dimensionality, as well as the exploratory approach used to identify potential associations with microbiota features.

## 3. Results

### 3.1. Characteristics of Participants

The analysis included 103 participants who had data available on dietary intake, microbiota composition, and intestinal permeability measures. The majority of the study participants were female (72.8%) with 64.1% self-reporting as African American and 32.0% reporting white race (Table 1). The average BMI was 37.5 ± 6.1 kg/m^2^. Characteristics of study participants based on their adherence to individual dietary patterns using the median score as cutoff are shown in Table 1. Median scores were lower in all three dietary patterns compared to maximum index scores (50.7 of a possible 100 [HEI-2010], 8.0 of a possible 21 [MDP], and 4.5 of a possible 11 [DASH]). Overall, participants in the HA group were more likely to be females and college graduates with a lower BMI compared to those in the LA group. For HEI-2010 and MDP, the HA group had lower metabolic syndrome scores relative to LA. Differences in total energy intake between HA and LA varied among dietary patterns.

### 3.2. Dietary Components

For HEI-2010, HA had higher intakes of healthy foods such as fruit, vegetables, and whole grain and lower intakes of less healthy foods/nutrients such as fat, refined grain and empty calorie foods compared to LA (*p* < 0.05). For MDP, the individual components that defined the differences between HA and LA were leafy greens, other vegetables, beans, and unsweetened beverages (*p* < 0.05), as well as olive oil and fish, components more unique to MDP (*p* < 0.05). For DASH, HA had higher intakes of total grain, whole grain, vegetables, fruit, and nuts and lower intakes of total fat and saturated fat compared to LA (*p* < 0.05). However, HA had higher sodium than LA (*p* = 0.02). Additionally, participants with HA had higher intakes of fruit and vegetable-related components in their diet as estimated by scores in HEI-2010 or by servings per week in the MDP and DASH dietary patterns, compared to those with LA across all three dietary patterns analyzed (Table 2).

### 3.3. Dietary Patterns and Intestinal Permeability

Higher scores of HEI-2010 (r = −0.33, *p* = 0.002), MDP (r = −0.31, *p* = 0.004) and DASH (r = −0.38, *p* < 0.0001) were associated with lower levels of 24 h urinary sucralose, a marker of total gut permeability. The three total dietary pattern scores were also inversely associated with 24 h sucralose-to-lactulose ratio, another marker for total gut permeability (HEI-2010: r = −0.23, *p* = 0.03; MDP: r = −0.32, *p* = 0.003; DASH: r = −0.24, *p* = 0.03) (Table 3). Comparison of adjusted means of outcome variables (permeability measures) between HA (≥median) and LA (<median) for each dietary pattern aligned with correlation analysis findings: overall, those in the HA group had lower 24 h urinary sucralose and 24 h sucralose-to-lactulose ratio compared to LA (*p* < 0.05). No associations were found for adherence to dietary pattern scores with other intestinal permeability measures. Since adherence to the above dietary patterns did not correlate with markers of small-intestinal permeability (5 h urinary mannitol or 5 h urinary lactulose), the observed inverse correlations with 24 h urinary sucralose and 24 h urinary sucralose-to-lactulose ratio suggest that “healthy” dietary patterns primarily impact colon permeability (Table 3).

To further understand how key components within dietary patterns influence intestinal permeability, associations between key permeability measures (24 h urinary sucralose and 24 h sucralose-to-lactulose ratio) and fruit and vegetable-related components were assessed (Table 4). These dietary components were chosen because their intakes differed significantly between HA and LA groups across all dietary patterns. Higher consumption of fruits (categorized as fruit, whole fruits, berries, and other fruits dependent on dietary pattern) were associated with lower permeability measures (24 h urinary sucralose and the 24 h sucralose-to-lactulose ratio, *p* < 0.05) across all three dietary patterns. In contrast, limited significant associations were found between intake of vegetable components and intestinal permeability measures except for “greens and beans” in the HEI-2010 dietary pattern.

### 3.4. Dietary Patterns and Microbiota Features

**Figure 1:**
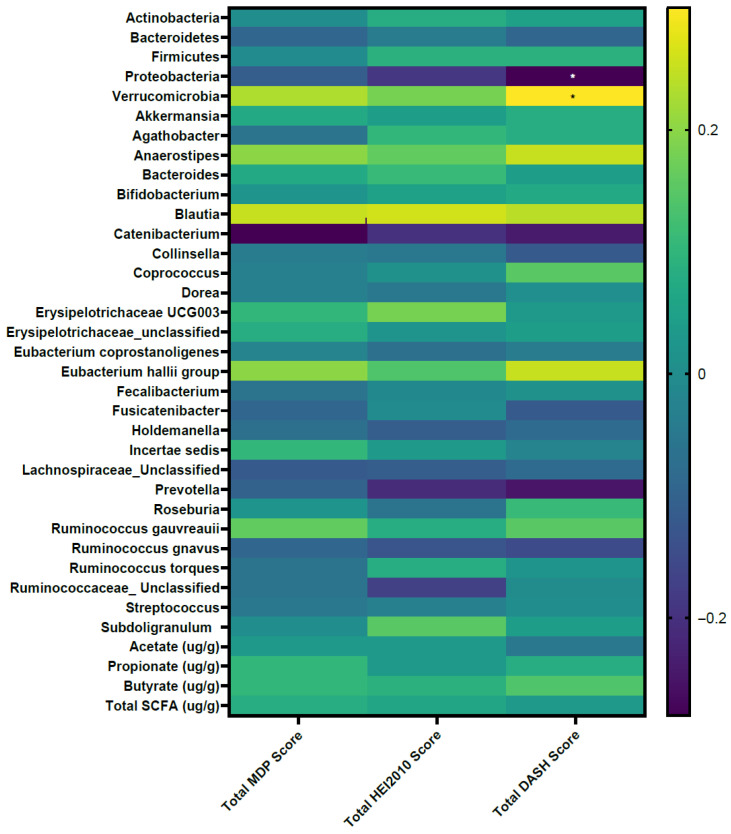
Correlations between adherence to dietary pattern scores and gut microbiota features. Correlation coefficient (r) and *p* values were determined using Spearman’s rank correlation coefficient and shading indicates positive (yellow) or negative (blue) associations. MDP = Mediterranean Dietary Pattern; HEI-2010 = Healthy Eating Index for 2010 dietary guidelines; DASH = Dietary Approaches to Stop Hypertension; SCFA = short-chain fatty acids. DASH was inversely correlated with Proteobacteria and positively correlated with Verrucomicrobia phylum abundance after adjusting for false discovery rate (*p* < 0.05) and indicated by *.

Correlations of adherence to dietary pattern scores with gut microbiota features are shown in Figure 1 and Supplementary Table S1. No associations were found between dietary pattern scores and both microbiota diversity (Shannon index, Simpson index) (*p* > 0.05) and fecal SCFA levels (*p* > 0.05). At the phylum level, DASH scores were inversely correlated with Proteobacteria abundance (r = −0.28, *p* = 0.004). Both DASH (r = 0.30, *p* = 0.002) and MDP (r = 0.23, *p* = 0.02) scores were positively correlated with Verrucomicrobia abundance. The associations of DASH with Proteobacteria and Verrucomicrobia abundance remained significant after adjustment for FDR (*p* < 0.05; Figure 1). At the genus level, adherence to dietary pattern scores were positively associated with *Blautia* abundance (HEI-2010: r = 0.26, *p* = 0.01; MDP: r = 0.25, *p* = 0.01; DASH: r = 0.24, *p* = 0.02), and inversely associated with *Catenibacterium* (HEI-2010: r = −0.20, *p* = 0.04; MDP: r = −0.28, *p* = 0.006; DASH: r = −0.24, *p* = 0.02) and *Prevotella* (HEI-2010: r = −0.21, *p* = 0.04, DASH: r = −0.25, *p* = 0.01) abundance. However, after FDR was applied, none of the above associations at the genus level were significant (*p* > 0.05).

### 3.5. Intestinal Permeability and Microbiota Features

**Figure 2:**
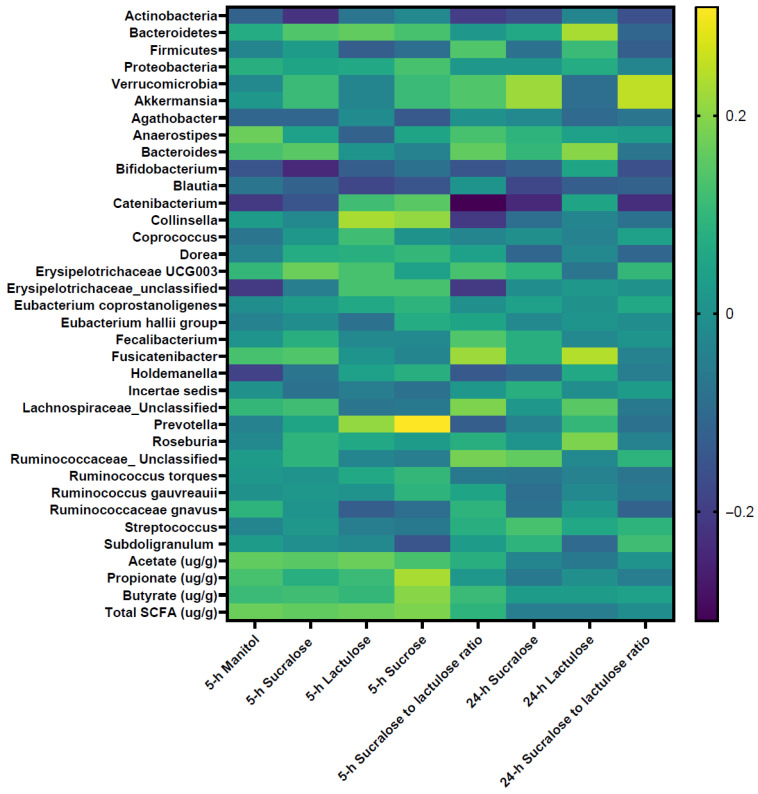
Correlations between intestinal permeability measures and gut microbiota features. Correlation coefficient (r) and *p* values were determined using Spearman’s rank correlation coefficient and are shaded to indicate positive (yellow) or negative (blue) associations. MDP = Mediterranean Dietary Pattern; HEI-2010 = Healthy Eating Index for 2010 dietary guidelines; DASH = Dietary Approaches to Stop Hypertension; SCFA = short-chain fatty acids. Correlations were not statistically significant after adjusting for false discovery rate (*p* > 0.05).

Figure 2 and Supplementary Table S2 present correlations of intestinal permeability measures with microbiota features. Permeability markers, 24 h sucralose (r = 0.22, *p* = 0.03) and 24 h sucralose-to-lactulose ratio (r = 0.25, *p* = 0.016) were positively correlated with the Verrucomicrobia phylum. Various correlations were also found at genus level. For example, 24 h sucralose (r = 0.22, *p* = 0.04) and 24 h sucralose-to-lactulose ratio (r = 0.25, *p* = 0.02) were positively correlated with *Akkermansia* (a mucus-degrading bacterium and a genus member of the Verrucomicrobia phylum) abundance. Nevertheless, none of the correlations between intestinal permeability measures and microbiota features were statistically significant after FDR correction (*p* > 0.05). No associations were found for permeability measures with diversity (Shannon index, Simpson index) and SCFA levels (*p* > 0.05).

## 4. Discussion

Given the limited research directly comparing adherence levels across the dietary patterns, it is important to consider how adherence was defined, as using the median to classify high and low accordance resulted in some individuals in the high group still having relatively low adherence scores. HEI scores above 80 are typically considered “good” scores while scores between 51 and 80 are considered in need of overall diet improvement [27]. Considering our HA average score of 65.5, our HA sample is not considered to be highly accordant to HEI. Similarly, the average MDP HA score of 10.3 in our sample aligned with the second tertile (10–12) of accordance in the original research validating the MDP screener [28]. While limited data exists for a similar age group and time period related to average DASH scores using the Folsom scoring method, given our average high accordance score of 6.1 out of 11 (55.5%), the HA group also had overall low accordance to the DASH diet when applying previous scoring interpretation methodologies [29]. These comparisons are crucial to interpreting our results as overall diet quality even in the HA group was relatively low, thus potentially limiting our ability to observe the benefits of a true HA to each of the three dietary patterns. However, the observed diet quality may reflect the typical characteristics of our study population, which consisted of individuals with obesity. Previous research has shown that low adherence to healthy dietary patterns, such as the Mediterranean diet, is associated with a higher prevalence of obesity [30,31].

The primary focus of the current study was to assess the associations of adherence to three healthful dietary patterns with intestinal permeability, given the limited research on this topic. Despite the sub-optimal adherence to all three dietary patterns, our results suggest that higher scores of adherence to healthful dietary patterns such as HEI-2010, MDP, and DASH were associated with lower intestinal permeability, as evidenced by lower levels of 24 h urinary sucralose and 24 h sucralose-to-lactulose ratio, primary indicators for total gut permeability [23]. The impact appears to be localized primarily to the colon, where the majority of gut bacteria reside. This is supported by significant correlations with 24 h urinary sucralose and the sucralose-to-lactulose ratio, whereas no significant associations were observed with 5 h urinary mannitol or lactulose, markers of small-intestinal permeability.

Research evidence suggests that obesity is often associated with increased intestinal permeability [13]. Therefore, improving diet quality through greater adherence to healthy dietary patterns may be particularly relevant for individuals with obesity, a notion supported by the findings observed in our study population. One important consideration is that there were differences between the HA and LA groups in key confounders including sex distribution, education level, BMI, and metabolic syndrome status, all of which could potentially influence the observed inverse associations between adherence to healthy dietary patterns and intestinal permeability. However, our findings were adjusted for relevant confounders, including age, sex, race/ethnicity, education, BMI, smoking status, alcohol consumption, total daily energy intake, and metabolic syndrome score.

In addition, our results show that those with high adherence to healthful dietary patterns had significantly higher intakes of fruits and vegetables compared to those with low adherence across all three dietary patterns. Intake of whole grains among HEI and DASH was also significantly higher compared to those with low diet adherence amongst those two diets. Thus, the overlap in food components such as fruits, vegetables, and whole grains among these dietary patterns may in part explain the potential beneficial associations between diet quality and reduced intestinal permeability observed across all three healthful dietary patterns. Higher ingestion of fruits, vegetables, and whole grains results in higher consumption of dietary fiber and polyphenols, both known to beneficially impact intestinal barrier function [32,33,34]. The antioxidant properties of polyphenols reduce oxidative stress which is known to weaken the tight junctions within the lining of the gut, thereby negatively impacting intestinal permeability [35]. In addition, polyphenols alleviate inflammation by decreasing levels of pro-inflammatory cytokines which have also been linked to a disruption in the tight junctions found within the lining [36]. In our study, we observed that higher fruit intake was consistently linked to lower intestinal permeability, as measured by 24 h urinary sucralose and the sucralose-to-lactulose ratio, across all dietary patterns. Fruits contain both insoluble and soluble fiber; however, many fruits—particularly citrus fruits—have a higher proportion of soluble fiber (e.g., pectin), which is readily fermented in the colon. Certain fruits, such as apples and pears, are also relatively high in fructose. Free fructose is poorly absorbed and may function similarly to dietary fiber by reaching the large intestine, where it undergoes fermentation. In addition, fruits are often highlighted as important sources of antioxidants, including vitamin C, polyphenols, and flavonoids [37]. Thus, the potential impact of fruit consumption on intestinal permeability, as suggested by our findings, may be attributable to its specific nutrient profile. Vegetables, particularly broccoli, carrots, cabbage, and leafy greens, tend to be richer in insoluble fiber [37]. Although we did not observe clear associations between overall vegetable intake scores and intestinal permeability, we did observe an inverse association with green vegetables and beans within HEI-2010. Thus, more detailed categorization of vegetable subgroups, along with more comprehensive dietary assessment approaches such as combining food frequency questionnaires with multiple 24 h dietary recalls or dietary records, may help clarify the relationship between vegetable intake and intestinal permeability.

The observed inverse associations between adherence to healthful dietary patterns and intestinal permeability suggest translational potential for dietary guidance in clinical and public health settings. Specifically, promoting increased consumption of fruits, vegetables, and whole grains—key components shared across HEI-2010, MDP, and DASH—may represent a practical strategy for improving gut barrier integrity, particularly among individuals with obesity. However, the scope of the current study, including its cross-sectional design and relatively limited sample size, highlights the need for expanded experimental approaches. Future research should include larger and more diverse study populations and employ longitudinal and randomized controlled trial designs to better evaluate the effects of dietary interventions, such as the Mediterranean diet, DASH diet, and other healthful dietary patterns, on gut health-related outcomes. In addition, more detailed dietary characterization including differentiation of fruit and vegetable types, fiber subtypes, and polyphenol profiles would improve interpretability and support the development of more precise and practical dietary recommendations.

In our study, we observed that DASH scores were inversely correlated with Proteobacteria phylum abundance (putative pro-inflammatory pathobiont) and positively correlated with the abundance of the Verrucomicrobia phylum. Evidence suggests that a higher abundance of Proteobacteria can disrupt the balance of the gut microbial community in humans, leading to dysbiosis [38]. This imbalance has been linked to metabolic disorders including type 2 diabetes, non-alcoholic fatty liver diseases, and atherosclerosis [39] and other systemic inflammatory disorders like inflammatory bowel disease [40]. In contrast, Verrucomicrobia has been implicated in supporting glucose homeostasis in the human gut [41] and exhibiting anti-inflammatory properties [42]. At genus level, the relationships between dietary pattern scores and microbiota features were not clear in our study. There were various associations detected; however, after FDR was applied, none of the associations were statistically significant, likely due to the relatively small sample size. Previous research has suggested that gut microbial composition differs between obese and non-obese individuals [16]. While all participants in our study were obese, we did not collect data on the duration of obesity or percent body fat. Because both the length of time an individual has been obese and their body fat percentage may influence gut and overall health, these factors should be assessed in future studies.

We also explored the relationships between intestinal permeability measures and gut microbiota features to determine whether any observed associations between adherence to healthful dietary patterns and intestinal permeability could be partially explained by the gut microbiota composition. Although there were a few associations detected between permeability measures and some microbiota features, these associations were not statistically significant after adjusting for FDR. Therefore, the relationships between intestinal permeability and gut microbiota were not conclusive based on the current results. Future studies are needed to more thoroughly explore the complex interrelationships among dietary patterns, intestinal permeability, and gut microbiota composition.

Our study is the first to examine relationships between adherence to healthful dietary patterns, intestinal permeability, and gut microbiota features in an obese sample. There are several limitations of our study. Due to the cross-sectional study design, we were unable to determine temporal sequence or directionality among diet, gut microbiota, and intestinal permeability, which limits conclusions about potential causal or mediating relationships. However, it is unlikely that outcomes (intestinal permeability, gut microbiota) would influence the exposure (adherence to dietary patterns). The relatively small sample size may limit our ability to detect statistically significant associations, particularly within bacteria genera after results were corrected for FDR. Since we utilized the baseline data of a randomized, controlled trial, which did not account for sampling strategy, our results may not be generalized. Additionally, a self-report food frequency questionnaire was used to estimate participants’ dietary intake and determine their adherence to the three heathy dietary patterns. Thus, we may not truly capture participants’ diet quality. With that said, the VioScreen food frequency questionnaire was validated, and its accuracy was determined previously [17]. The use of median-based dichotomization may have contributed to relatively low absolute diet quality in the high-adherence group (HA), potentially limiting the interpretation of the current findings. Lastly, HEI-2010, MPD and DASH did not include a distinct component for processed foods, which may have introduced bias in the construction of the diet quality scores.

## 5. Conclusions

Our results indicate that adherence to healthful dietary patterns such as HEI-2010, MDP, and DASH was associated with reduced intestinal permeability as evidenced by decreased levels of 24 h urinary sucralose and the sucralose-to-lactulose ratio in those with high adherence. Among dietary components, fruit intake appeared to play a more relevant role in relation to reduced intestinal permeability. DASH scores were inversely correlated with abundance of the pathobiont Proteobacteria and positively correlated with Verrucomicrobia abundance. The relationships between intestinal permeability and gut microbiota features were not conclusive in the study. Overall, these findings suggest diet quality reflected by adherence to healthful dietary patterns like HEI-2010, MDP, and DASH may positively impact gut health in individuals with obesity. However, due to the cross-sectional study design of the current investigation, the potential impact of diet quality and dietary patterns on intestinal permeability and gut microbiota features needs to be further assessed through rigorously designed clinical trials.

## Figures and Tables

**Table 1:** Characteristics of study participants by adherence to individual dietary patterns.

	All Participants	HEI-2010 Median = 59.7		MDP Median = 8.0		DASH Median = 4.5	
		LA (<59.7)	HA (≥59.7)	LA (<8.0)	HA (≥8.0)	LA (<4.5)	HA (≥4.5)
N	103	51	52	48	55	43	60
Dietary index, mean (range)		49.1 (25.8–59.6)	68.9 (59.7–87.7)	5.5 (2.0–7.0)	10.1 (8.0–15.0)	3.1 (1.0–4.0)	6.0 (4.5–9.5)
Age (Year)	43.8 ± 11.3	42.5 ± 11.0	45.0 ± 11.6	42.4 ± 11.5	45.0 ± 11.2	43.5 ± 12.0	44.0 ± 10.9
Sex, N (%)							
Male	28 (27.2)	18 (35.3)	10 (19.2)	18 (37.5) *	10 (18.2) *	13 (30.2)	15 (25.0)
Female	75 (72.8)	33 (64.7)	42 (80.7)	30 (62.5) *	45 (81.8) *	30 (69.8)	45 (75.0)
Race							
African American	66 (64.1)	36 (70.6)	30 (57.7)	30 (62.5)	36 (65.5)	29 (67.4)	37 (61.7)
White	33 (32.0)	14 (27.4)	19 (36.5)	16 (31.3)	17 (30.9)	13 (30.2)	20 (33.3)
Other	4 (3.9)	1 (2.0)	3 (5.8)	2 (4.2)	2 (3.6)	1 (2.3)	3 (5.0)
College or above, N (%)	41 (68.2)	13 (25.5) *	28 (53.8) *	16 (33.4)	25 (45.5)	16 (37.2)	25 (41.7)
BMI (kg/m^2^)	37.5 ± 6.1	38.9 ± 6.5 *	36.2 ± 5.3 *	38.2 ± 5.9	36.9 ± 6.1	38.2 ± 6.0	37.0 ± 6.1
Current smoker, N (%)	18 (17.6)	11 (22.0)	7(13.5)	9 (18.8)	9 (16.4)	6 (14.3)	12 (20.0)
Alcohol use, N (%)							
Non-user	36 (35.0)	17 (33.4)	19 (36.5)	19 (39.6)	17 (30.9)	16 (37.2)	20 (33.3)
Rare user	21 (20.4)	8 (15.6)	13 (25.1)	4 (8.3) *	17 (30.9) *	7 (16.3)	14 (23.3)
Occasional user	19 (18.4)	13 (25.5)	6 (11.5)	11 (22.9)	8 (14.6)	9 (20.9)	10 (16.7)
≥1 drink/week	27 (26.2)	13 (25.5)	14 (26.9)	14 (29.2)	13 (23.6)	11 (25.6)	16 (26.7)
Total energy intake (kcal)	2902 ± 2654	3577 ± 3155 *	2241 ± 1851 *	2830 ± 1811	2965 ± 3232	2300 ± 1446 *	3334 ± 3200 *
Metabolic syndrome score ^a^	2.2 ± 1.2	2.5 ± 1.2 *	1.8 ± 1.1 *	2.6 ± 1.1 *	1.9 ± 1.2 *	2.3 ± 1.2	2.1 ± 1.2

Data are given as mean ± standard deviation unless otherwise specified. Abbreviations: LA = low adherence to the specific dietary pattern; HA = high adherence to the specific dietary pattern as defined by median intake; HEI-2010 = Healthy Eating Index for 2010 dietary guidelines; MDP = Mediterranean Dietary Pattern; DASH = Dietary Approaches to Stop Hypertension; BMI = body mass index. * Values were statistically significantly different (*p* < 0.05) within each dietary pattern category between high- and low-adherence status using *t* test for continues variables and chi-square test for categorical variables. ^a^ Maximum metabolic syndrome score of 5 (metabolic components: high waist circumference, high triglyceride level, reduced HDL, increased blood pressure, and elevated fasting blood sugar).

**Table 2:** Intake of individual dietary components by adherence to dietary pattern.

	LA ^a^	HA ^a^	*p* ^b^
**HEI-2010 component score**			
Fruit	2.13 ± 1.45	3.84 ± 1.44	<0.001
Whole fruit	2.16 ± 1.46	4.00 ± 1.35	<0.001
Vegetables	3.29 ± 1.07	4.28 ± 1.14	<0.001
Greens and beans	1.69 ± 1.40	3.90 ± 1.37	<0.001
Whole grains	3.07 ± 2.21	5.61 ± 3.20	<0.001
Dairy	5.81 ± 2.40	6.16 ± 2.83	0.50
Protein foods	4.27 ± 0.97	4.57 ± 0.91	0.11
Seafoods and plant protein	2.87 ± 1.66	3.75 ± 1.69	0.009
Fatty acid ^c^	4.23 ± 2.54	5.99 ± 3.13	0.002
Refined grains ^d^	7.17 ± 3.05	8.80 ± 2.43	0.004
Sodium ^d^	3.22 ± 3.10	2.16 ± 2.43	0.055
Empty calories ^d^	9.17 ± 4.01	15.88 ± 3.17	<0.001
**MDP component (serving/week)**			
Olive oil	0.90 ± 1.56	2.59 ± 5.65	0.037
Butter/cream	4.90 ± 8.16	3.36 ± 7.30	0.31
Nuts	1.72 ± 3.33	2.82 ± 3.74	0.12
Avocado	0.38 ± 1.13	3.08 ± 8.13	0.18
Berries	0.61 ± 1.12	2.10 ± 3.16	0.002
Other fruit	9.77 ± 10.05	24.99 ± 38.99	0.007
Leafy greens	2.67 ± 5.19	6.52 ± 6.31	<0.001
Other vegetables	8.63 ± 5.17	19.52 ± 25.00	0.001
Red/processed meat	8.99 ± 9.07	6.77 ± 10.79	0.26
Poultry	2.64 ± 2.76	3.71 ± 5.19	0.19
Fish	0.66 ± 1.17	3.33 ± 6.36	0.003
Milk/yogurt	5.33 ± 6.41	7.30 ± 14.22	0.38
Cheese	3.86 ± 4.61	2.77 ± 4.21	0.22
Beans	0.59 ± 1.09	3.84 ± 9.33	<0.0001
Sweets	13.40 ± 9.66	12.33 ± 24.10	0.77
Fast food	4.39 ± 3.66	2.94 ± 3.68	0.049
Prepackaged food	2.51 ± 2.18	2.48 ± 3.88	0.96
Sweetened beverages	10.02 ± 10.92	10.06 ± 23.52	0.99
Whole grains	9.25 ± 9.03	14.60 ± 19.43	0.08
Alcohol	7.86 ± 17.12	3.69 ± 6.63	0.12
Unsweetened beverages	27.70 ± 17.94	41.30 ± 22.68	0.001
**DASH components (serving/week)**			
Total grain	35.23 ± 20.52	79.64 ± 70.58	<0.001
Whole grain	8.50 ± 5.24	26.62 ± 27.52	<0.001
Vegetable	8.50 ± 5.25	26.62 ± 27.52	<0.001
Fruit	9.06 ± 9.48	29.68 ± 44.89	<0.001
Dairy	12.24 ± 11.04	18.01 ± 22.96	0.09
Meat	19.24 ± 15.46	19.83 ± 20.91	0.88
Nuts/seeds/beans	2.19 ± 4.25	10.00 ± 21.84	0.009
Total fat (% total kcal)	38.24 ± 6.39	32.60 ± 6.98	<0.001
Saturated fat (% total kcal)	12.58 ± 2.39	10.36 ± 3.22	<0.001
Sweets	17.76 ± 13.39	22.44 ± 41.57	0.48
Sodium (g/d)	4.07 ± 2.69	6.08 ± 5.94	0.02

Data presented as mean ± standard deviation. Abbreviations: HEI-2010 = Healthy Eating Index for 2010 dietary guidelines; MDP = Mediterranean Dietary Pattern for Americans; DASH = Dietary Approaches to Stop Hypertension. ^a^ HEI-2010: low adherence, <median score (<59.7); high adherence, ≥median sore (≥59.7). MDP: low adherence, <median score (<8.0); high adherence, ≥median sore (≥8.0). DASH: low adherence, <median score (<4.5); high adherence, ≥median sore (≥4.5). Maximum scores: HEI 2010 score 100, MDP score 21, DASH score 11. ^b^ *p* values for differences between low-adherence vs. high-adherence groups of individual dietary patterns using *t* test. ^c^ Ratio of the sum of monounsaturated and polyunsaturated fatty acids to saturated fatty acids. ^d^ Reverse-scored.

**Table 3:** Correlation between dietary patterns and intestinal permeability.

	HEI-2010				MDP				DASH			
Permeability ^a^	r (*p*) ^b^	LA (n = 46)	HA (n = 50)	*p* ^c^	r (*p*) ^b^	LA (n = 45)	HA (n = 51)	*p* ^c^	r (*p*) ^b^	LA (n = 39)	HA (n = 57)	*p* ^c^
5 h mannitol	0.11 (0.32)	10.84 ± 6.96	11.34 ± 7.29	0.72	−0.05 (0.68)	11.03 ± 6.44	11.16 ± 7.70	0.89	0.04 (0.73)	9.67 ± 6.37	12.07 ± 7.46	0.12
5 h sucralose	−0.08 (0.46)	0.30 ± 0.26	0.27 ± 0.30	0.50	−0.20 (0.06)	0.32 ± 0.25	0.25 ± 0.30	0.13	−0.06 (0.60)	0.24 ± 0.18	0.31 ± 0.33	0.37
5 h lactulose	0.05 (0.64)	0.90 ± 0.99	0.78 ± 0.74	0.83	0.04 (0.75)	0.82 ± 0.58	0.86 ± 1.07	0.48	−0.01 (0.94)	0.68 ± 0.36	0.95 ± 1.08	0.12
5 h sucrose	0.06 (0.61)	0.37 ± 0.25	0.36 ± 0.43	0.62	0.03 (0.79)	0.35 ± 0.22	0.38 ± 0.44	0.49	0.03 (0.80)	0.27 ± 0.14	0.42 ± 0.43	0.02
5 h sucralose-to-lactulose ratio	−0.12 (0.27)	0.37 ± 0.24	0.42 ± 0.38	0.56	−0.24 (0.27)	0.42 ± 0.27	0.38 ± 0.36	0.04	−0.06 (0.60)	0.38 ± 0.32	0.41 ± 0.33	0.96
24 h sucralose	−0.33 (0.002)	0.91 ± 0.75	0.67 ± 0.45	0.12	−0.38 (<0.0001)	0.92 ± 0.72	0.67 ± 0.50	0.04	−0.31 (0.004)	0.91 ± 0.64	0.70 ± 0.60	0.02
24 h lactulose	−0.10 (0.35)	3.68 ± 2.28	3.40 ± 2.14	0.92	−0.02 (0.84)	3.58 ± 1.74	3.49 ± 2.54	0.77	−0.06 (0.59)	3.36 ± 1.27	3.66 ± 2.69	0.72
24 h sucralose-to-lactulose ratio	−0.23 (0.03)	0.27 ± 0.18	0.25 ± 0.20	0.14	−0.32 (0.003)	0.27 ± 0.18	0.25 ± 0.20	0.04	−0.24 (0.03)	0.29 ± 0.19	0.24 ± 0.19	0.02

Abbreviations: HEI-2010 = Healthy Eating Index for 2010 dietary guidelines; MDP = Mediterranean Dietary Pattern; DASH = Dietary Approaches to Stop Hypertension. ^a^ Permeability was determined by the percentage of ingested oral dosage of a sugar mixture (mannitol, lactulose, sucrose, and sucralose) excreted in the urine. ^b^ Partial correlation coefficient (r) and *p* value adjusting for age, sex, race/ethnicity, education, body mass index, smoking status, daily total energy intake, frequency of alcohol consumption and metabolic syndrome score. Log-transformed values of permeability biomarkers were used to compute r and *p* values, ^c^ *p* values for differences between participants by low adherence to individual dietary pattern (LA; score < median) and high adherence to individual dietary pattern (HA, score ≥ median) using GLM adjusting for age, sex, race/ethnicity, education, body mass index, smoking status, daily total energy intake, frequency of alcohol consumption and metabolic syndrome score. Log-transformed values of permeability biomarkers were used to compute *p* values.

**Table 4:** Associations of relevant dietary components with key intestinal permeability measures.

HEI-2010 (Scores)																
	Fruit				Whole Fruits				Vegetables				Greens and Beans			
Permeability	r (*p*) ^a^	<Median	≥Median	*p* ^b^	r (*p*) ^a^	<Median	≥Median	*p* ^b^	r (*p*) ^a^	<Median	≥Median	*p* ^b^	r (*p*) ^a^	<Median	≥Median	*p* ^b^
24 h sucralose	−0.40 (<0.001)	1.01 ± 0.74	0.32 ± 0.22	<0.001	−0.27 (0.01)	0.91 ± 0.69	0.66 ± 0.52	0.10	−0.14 (0.20)	0.90 ± 0.67	0.67 ± 0.55	0.02	−0.26 (0.02)	0.94 ± 0.74	0.63 ± 0.44	0.02
24 h sucralose-to-lactulose ratio	−0.34 (0.001)	0.27 ± 0.18	0.20 ± 0.14	0.002	−0.18 (0.11)	0.28 ± 0.21	0.24 ± 0.15	0.58	−0.07 (0.52)	0.27 ± 0.18	0.25 ± 0.19	0.12	−0.21 (0.05)	0.28 ± 0.20	0.24 ± 0.18	0.15
**MDP (Serving/Week)**																
	Berries				Other Fruits				Leafy Green Vegetables				Other Vegetables			
	r (*p*) ^a^	<Median	≥Median	*p* ^b^	r (*p*) ^a^	<Median	≥Median	*p* ^b^	r (*p*) ^a^	<Median	≥Median	*p* ^b^	r (*p*) ^a^	<Median	≥Median	*p* ^b^
24 h sucralose	−0.29 (0.008)	0.86 ± 0.67	0.67 ± 0.49	0.12	−0.35 (0.001)	0.89 ± 0.67	0.67 ± 0.55	0.03	−0.14 (0.20)	0.84 ± 0.67	0.73 ± 0.56	0.56	−0.15 (0.18)	0.78 ± 0.61	0.79 ± 0.65	0.57
24 h sucralose-to-lactulose ratio	−0.21 (0.05)	0.26 ± 0.20	0.25 ± 0.17	0.58	−0.30 (0.006)	0.29 ± 0.22	0.23 ± 0.15	0.10	−0.14 (0.20)	0.27 ± 0.20	0.24 ± 0.18	0.32	−0.10 (0.35)	0.26 ± 0.19	0.26 ± 0.19	0.66
**DASH (Serving/Week)**																
	Fruit				Vegetables											
	r (*p*) ^a^	<Median	≥Median	*p* ^b^	r (*p*) ^a^	<Median	≥Median	*p* ^b^								
24 h sucralose	−0.35 (0.001)	0.90 ± 0.67	0.67 ± 0.55	0.017	−0.16 (0.15)	0.84 ± 0.69	0.73 ± 0.55	0.25								
24 h sucralose-to-lactulose ratio	−0.28 (0.009)	0.29 ± 0.21	0.23 ± 0.16	0.057	−0.12 (0.28)	0.28 ± 0.22	0.24 ± 0.14	0.32								

Each dietary pattern is assessed using a distinct scoring method based on specific groupings of fruits and vegetables. Abbreviations: HEI-2010 = Healthy Eating Index for 2010 dietary guidelines; MDP = Mediterranean Dietary Pattern; DASH = Dietary Approaches to Stop Hypertension. ^a^ Partial correlation coefficient (r) and *p* value adjusting for age, sex, race/ethnicity, education, body mass index, smoking status, daily total energy intake, frequency of alcohol consumption and metabolic syndrome score. Log-transformed values of permeability biomarkers were used to compute r and *p* values, ^b^ *p* values for differences between participants with low intake of individual food component (<median) and participants with high intake of individual food component (≥median) using GLM adjusting for age, sex, race/ethnicity, education, body mass index, smoking status, daily total energy intake, frequency of alcohol consumption and metabolic syndrome score. Log-transformed values of permeability biomarkers were used to compute *p* values.

## Data Availability

Data supporting the reported results are included in the main tables and figures and Supplementary Tables.

## Supplementary Materials

The following supporting information can be downloaded at: https://www.mdpi.com/article/10.3390/nu18050775/s1, Table S1: Correlations between dietary patterns with gut microbiota features; Table S2: Associations between intestinal permeability and gut microbiota features.
