# Supplementary material for: Relationship Between Diet Quality, Intestinal Permeability, and Gut Microbiota Features in Individuals with Obesity
Source: Nutrients. 2026 Feb 27;18(5):775. doi: 10.3390/nu18050775 (PMC12987183; doi:10.3390/nu18050775)
Supplement: Supplementary file 1 [file nutrients-18-00775-s001.zip › nutrients-4136380-supplementary.pdf]

Supplementary Table S1. Correlations between dietary patterns with gut microbiota features

	HEI-2010 score r (*P*)[a]	MDP Score r (*P*)[a]	DASH Score r (*P*)[a]
**Phylum**			
Actinobacteria	0.08 (0.39)	0.001 (0.99)	0.05 (0.59)
Bacteroidetes	-0.04 (0.68)	-0.09 (0.37)	-0.09 (0.36)
Firmicutes	0.09 (0.37)	-0.003 (0.97)	0.09 (0.35)
Proteobacteria	-0.19 (0.06)	-0.11 (0.28)	**-0.28 (0.004)**[b] FDR: *P*=0.01
Verrucomicrobia	0.18 (0.07)	0.23 (0.02)	**0.30 (0.002)**[b] FDR: *P*=0.01
**Genus**			
Akkermansia	0.04 (0.68)	0.07 (0.47)	0.08 (0.45)
Agathobacter	0.10 (0.33)	-0.06 (0.55)	0.08 (0.42)
Anaerostipes	0.16 (0.11)	0.20 (0.048)	0.25 (0.01)
Bacteroides	0.11 (0.27)	0.07 (0.48)	0.04 (0.68)
Bifidobacterium	0.05 (0.61)	0.02 (0.81)	0.07 (0.48)
Blautia	0.26 (0.01)	0.25 (0.01)	0.24 (0.02)
Catenibacterium	-0.20 (0.04)	-0.28 (0.006)	-0.24 (0.02)
Collinsella	-0.05 (0.64)	-0.04 (0.67)	-0.12 (0.22)
Coprococcus	0.01(0.90)	-0.03 (0.79)	0.15 (0.13)
Dorea	-0.05 (0.59)	-0.03 (0.80)	0.005 (0.96)
Erysipelotrichaceae_UCG003	0.18 (0.07)	0.10 (0.33)	0.03 (0.74)
Erysipelotrichaceae_unclassified	0.02(0.84)	0.08 (0.42)	0.04 (0.72)
Eubacterium coprostanoligenes	-0.07 (0.46)	-0.02 (0.84)	-0.04 (0.70)
Eubacterium hallii group	0.14 (0.16)	0.20 (0.046)	0.25 (0.02)
Fecalibacterium	-0.01 (0.92)	-0.06 (0.53)	0.01(0.89)
Fusicatenibacter	-0.003 (0.98)	-0.09 (0.35)	-0.12 (0.25)
Holdemanella	-0.11 (0.27)	-0.07 (0.51)	-0.08 (0.40)
Incertae sedis	0.03(0.73)	0.10 (0.32)	-0.02 (0.81)
Lachnospiraceae_Unclassified	-0.11(0.28)	-0.12 (0.24)	-0.08 (0.42)
Prevotella	-0.21 (0.04)	-0.10 (0.32)	-0.25 (0.01)
Roseburia	-0.06 (0.54)	0.02 (0.85)	0.11 (0.29)
Ruminococcaceae_Unclassified	0.08 (0.43)	0.16 (0.11)	0.15 (0.13)
Ruminococcus torques	-0.13 (0.21)	-0.09 (0.40)	-0.15 (0.14)
Ruminococcus gauvreauii	0.08 (0.42)	-0.06 (0.51)	0.02(0.84)
Ruminococcus gnavus	-0.17 (0.08)	-0.06 (0.54)	-0.0006 (0.99)
Streptococcus	-0.03 (0.78)	-0.05 (0.62)	0.001 (0.99)
Subdoligranulum	0.15 (0.15)	0.001 (0.94)	0.03(0.67)
**SCFA**			
Acetate (ug/g)	0.03 (0.73)	0.03 (0.79)	-0.05 (0.67)
Propionate (ug/g)	0.03 (0.81)	0.10 (0.38)	0.08 (0.50)
Butyrate (ug/g)	0.09 (0.42)	0.10 (0.37)	0.14 (0.21)
Total SCFA (ug/g)	0.06 (0.56)	0.08 (0.49)	0.04 (0.77)

HEI-2010= healthy Eating Index for 2010 dietary guidelines; MDP = Mediterranean Dietary Pattern; DASH= Dietary Approaches to Stop Hypertension; SCFA = short chain fatty acid.

[a] Correlation coefficient (r) and *P* values were estimated by Spearman's Rank Correlation Coefficient analysis.

[b] Statistically significant after false discovery rate (FDR) correction.

Supplementary Table S2. Associations between intestinal permeability and gut microbiota features[a]

	5-h Mannitol	5-h Sucralose	5-h Lactulose	5-h Sucrose	5-hr Sucralose to lactulose ratio	24-h Sucralose	24-h Lactulose	24-h Sucralose to lactulose ratio
**Phylum**								
Actinobacteria	-0.12 (0.26)	-0.22 (0.03)[b]	-0.07 (0.52)	-0.02 (0.85)	-0.20 (0.05)	-0.17 (0.10)	-0.03 (0.76)	-0.16 (0.11)
Bacteroidetes	0.07 (0.50)	0.14 (0.16)	0.16 (0.13)	0.13 (0.51)	0.02 (0.83)	0.06 (0.60)	0.23 (0.03)[b]	-0.11 (0.29)
Firmicutes	-0.03 (0.81)	0.03 (0.75)	-0.13 (0.22)	-0.09 (0.38)	0.14 (0.17)	-0.08 (0.47)	0.11 (0.27)	-0.13 (0.21)
Proteobacteria	0.08 (0.45)	0.05 (0.64)	0.06 (0.55)	0.13 (0.22)	0.02 (0.87)	0.02 (0.88)	0.07 (0.50)	-0.03 (0.78)
Verrucomicrobia	-0.02 (0.83)	0.11 (0.31)	-0.03 (0.77)	0.11 (0.32)	0.14 (0.17)	0.22 (0.03)[b]	-0.09 (0.41)	0.25 (0.016)[b]
**Genus**								
Akkermansia	0.02 (0.83)	0.11 (0.31)	-0.03 (0.77)	0.11 (0.32)	0.14 (0.17)	0.22 (0.04)[b]	-0.09 (0.42)	0.25 (0.02)[b]
Agathobacter	-0.11 (0.31)	-0.11 (0.28)	-0.14(0.18)	-0.14 (0.18)	-0.001 (0.99)	-0.02 (0.82)	-0.10 (0.36)	-0.07 (0.52)
Anaerostipes	0.17 (0.11)	0.04 (0.69)	-0.12 (0.26)	-0.05 (0.63)	0.13 (0.21)	0.09 (0.37)	0.04 (0.68)	0.03 (0.75)
Bacteroides	0.13 (0.21)	0.15 (0.15)	0.01 (0.90)	-0.04 (0.72)	0.16 (0.13)	0.10 (0.35)	0.20 (0.06)	-0.07 (0.51)
Bifidobacterium	-0.15 (0.16)	-0.24 (0.02)[b]	-0.13 (0.23)	-0.08 (0.44)	-0.15 (0.15)	-0.12 (0.26)	0.05 (0.62)	-0.16 (0.13)
Blautia	-0.07 (0.49)	-0.12 (0.26)	-0.18 (0.08)	-0.15 (0.16)	0.01 (0.91)	-0.18 (0.09)	-0.13 (0.23)	-0.12 (0.27)
Catenibacterium	-0.21 (0.05)	-0.15 (0.17)	0.12 (0.24)	0.15 (0.17)	-0.31 (0.003)[b]	-0.24 (0.02)[b]	0.05 (0.64)	-0.23 (0.02)[b]
Collinsella	0.03 (0.79)	-0.02 (0.82)	0.23 (0.03)	0.21 (0.05)	-0.21 (0.04)[b]	-0.09 (0.39)	-0.03 (0.78)	-0.08 (0.44)
Coprococcus	-0.07 (0.47)	0.02 (0.84)	0.12 (0.25)	0.006 (0.95)	-0.03 (0.80)	-0.003 (0.98)	-0.04 (0.71)	0.04 (0.70)
Dorea	-0.04 (0.73)	0.07 (0.52)	0.08 (0.46)	0.10 (0.33)	0.04 (0.74)	-0.11 (0.28)	-0.02 (0.85)	-0.11 (0.30)
Erysipelotrichaceae UCG003	0.10 (0.36)	0.17 (0.11)	0.13 (0.20)	0.04 (0.74)	0.13 (0.22)	0.09 (0.42)	-0.07 (0.52)	0.10 (0.35)
Erysipelotrichaceae_unclassified	-0.21 (0.04)[b]	-0.05 (0.63)	0.13 (0.22)	0.13 (0.22)	-0.21 (0.04)[b]	-0.01 (0.90)	0.02 (0.82)	-0.001 (0.99)
Eubacterium coprostanoligenes	-0.007 (0.94)	0.03 (0.74)	0.06 (0.59)	0.09 (0.38)	-0.003 (0.98)	0.04 (0.68)	-0.01 (0.94)	0.06 (0.57)
Eubacterium hallii group	-0.04 (0.86)	-0.01 (0.92)	-0.08 (0.46)	0.07 (0.48)	0.05 (0.64)	-0.02 (0.87)	0.01 (0.91)	-0.01 (0.92)
Fecalibacterium	0.01 (0.89)	0.08 (0.45)	-0.02 (0.82)	-0.02 (0.86)	0.14 (0.18)	0.08 (0.42)	-0.02 (0.80)	0.01 (0.40)
Fusicatenibacter	0.13 (0.21)	0.14 (0.19)	0.01 (0.90)	-0.03 (0.75)	0.22 (0.04)[b]	0.08 (0.45)	0.24 (0.02)[b]	-0.04 (0.72)
Holdemanella	-0.19 (0.06)	-0.07 (0.50)	0.04 (0.72)	0.08 (0.47)	-0.14 (0.19)	-0.11 (0.28)	0.06 (0.59)	-0.05 (0.61)
Incertae sedis	-0.0001 (0.99)	-0.08 (0.46)	-0.05 (0.63)	-0.08 (0.47)	0.02 (0.97)	0.08 (0.45)	-0.008 (0.94)	0.03 (0.80)

Lachnospiraceae_ Unclassified	0.10 (0.35)	0.12 (0.24)	-0.07 (0.50)	-0.06 (0.54)	0.19 (0.06)	0.02 (0.87)	0.15 (0.16)	-0.06 (0.60)
Prevotella	-0.04 (0.70)	0.05 (0.62)	0.21 (0.04)	0.31 (0.003)[b]	-0.13 (0.21)	-0.04 (0.73)	0.10 (0.35)	-0.08 (0.45)
Roseburia	-0.02 (0.81)	0.09 (0.41)	0.06 (0.54)	0.03 (0.75)	0.08 (0.47)	0.01 (0.92)	0.19 (0.07)	-0.04 (0.72)
Ruminococcaceae family Unclassified	0.03 (0.77)	0.09 (0.42)	-0.03 (0.75)	-0.05 (0.63)	0.18 (0.09)	0.16 (0.14)	-0.02 (0.92)	0.09 (0.37)
Ruminococcus torques	0.02 (0.88)	0.007 (0.95)	0.06 (0.55)	0.10 (0.37)	-0.06 (0.56)	-0.07 (0.53)	-0.04 (0.72)	-0.07 (0.53)
Ruminococcus gauvreauii	0.001 (0.99)	0.02 (0.88)	0.007 (0.94)	0.09 (0.42)	0.05 (0.62)	-0.09 (0.40)	-0.02 (0.87)	-0.06 (0.54)
Ruminococcus gnavus	0.09 (0.41)	0.01 (0.89)	-0.13 (0.22)	-0.09 (0.40)	0.09 (0.42)	-0.08 (0.47)	0.02 (0.84)	-0.12 (0.26)
Streptococcus	-0.03 (0.74)	0.02 (0.89)	-0.05 (0.63)	-0.06 (0.64)	0.08 (0.46)	0.13 (0.23)	0.06 (0.56)	0.09 (0.40)
Subdoligranulum	0.03 (0.74)	-0.005 (0.96)	-0.02 (0.81)	-0.15 (0.18)	0.03 (0.79)	0.09 (0.42)	-0.10 (0.33)	0.12 (0.24)
**SCFA**[c]								
Acetate (ug/g)	0.16 (0.16)	0.15 (0.20)	0.17 (0.15)	0.13 (0.27)	0.08 (0.49)	-0.03 (0.77)	-0.06 (0.62)	0.008 (0.95)
Propionate (ug/g)	0.13 (0.28)	0.08 (0.47)	0.11 (0.34)	0.23 (0.05)	0.02 (0.84)	-0.06 (0.62)	-0.003 (0.97)	-0.05 (0.64)
Butyrate (ug/g)	0.11 (0.33)	0.12 (0.31)	0.10 (0.39)	0.20 (0.09)	0.11 (0.36)	0.03 (0.81)	0.03 (0.83)	0.04 (0.73)
Total SCFA (ug/g)	0.17 (0.15)	0.16 (0.17)	0.17 (0.15)	0.19 (0.11)	0.09 (0.45)	-0.05 (0.64)	-0.05 (0.66)	-0.008 (0.95)

[a] Data are presented as r (*P* value). Correlation coefficient (r) and *P* values are estimated by Spearman Rank Correlation Coefficient analysis.
[b] ***P* values were not statistically significant (*P*>0.05) after adjusting for False discover rate (FDR).**
All the correlations between intestinal permeability markers and microbiota features were not statistically significant after adjusting for FDR (*P*>0.05).
[c] SCFA = short chain fatty acids
